# Model-based classification of CPT data and automated lithostratigraphic mapping for high-resolution characterization of a heterogeneous sedimentary aquifer

**DOI:** 10.1371/journal.pone.0176656

**Published:** 2017-05-03

**Authors:** Bart Rogiers, Dirk Mallants, Okke Batelaan, Matej Gedeon, Marijke Huysmans, Alain Dassargues

**Affiliations:** 1 Institute for Environment, Health and Safety, Belgian Nuclear Research Centre (SCK•CEN), Mol, Belgium; 2 CSIRO Land and Water, Glen Osmond, South Australia, Australia; 3 School of the Environment, Flinders University, Adelaide, South Australia, Australia; 4 Dept. of Earth and Environmental Sciences, KU Leuven, Heverlee, Belgium; 5 Dept. of Hydrology and Hydraulic Engineering, Vrije Universiteit Brussel, Brussels, Belgium; 6 Hydrogeology and Environmental Geology, Dept. of Architecture, Geology, Environment and Civil Engineering (ArGEnCo) and Aquapole, Université de Liège, Liège, Belgium; Tianjin University, CHINA

## Abstract

Cone penetration testing (CPT) is one of the most efficient and versatile methods currently available for geotechnical, lithostratigraphic and hydrogeological site characterization. Currently available methods for soil behaviour type classification (SBT) of CPT data however have severe limitations, often restricting their application to a local scale. For parameterization of regional groundwater flow or geotechnical models, and delineation of regional hydro- or lithostratigraphy, regional SBT classification would be very useful. This paper investigates the use of model-based clustering for SBT classification, and the influence of different clustering approaches on the properties and spatial distribution of the obtained soil classes. We additionally propose a methodology for automated lithostratigraphic mapping of regionally occurring sedimentary units using SBT classification. The methodology is applied to a large CPT dataset, covering a groundwater basin of ~60 km^2^ with predominantly unconsolidated sandy sediments in northern Belgium. Results show that the model-based approach is superior in detecting the true lithological classes when compared to more frequently applied unsupervised classification approaches or literature classification diagrams. We demonstrate that automated mapping of lithostratigraphic units using advanced SBT classification techniques can provide a large gain in efficiency, compared to more time-consuming manual approaches and yields at least equally accurate results.

## Introduction

Cone penetration testing is one of the most efficient and versatile methods currently available for geotechnical and stratigraphic site characterization [1]. After being developed at the Dutch Laboratory for Soil Mechanics in Delft [2], its use for soil investigation quickly spread worldwide. While the family of direct push methods has known a great expansion during the last decade [3], standard cone penetration tests (CPTs), possibly extended with pore pressure logging (CPTu), are still the most widely used techniques. Due to the maturity of these methods, their speed, cost, precision, accuracy, and repeatability are unmatched today.

The classical interpretations of standard CPTs in geotechnical literature are performed by visual examination of the raw data or the use of empirical soil (or soil behaviour type—SBT) classification charts [4, 5, 1]. More recent work in the framework of interpretation or classification of CPT data is mostly focussed on using Bayesian approaches [6, 7], fuzzy classification techniques [8, 9], hierarchical and *k*-means clustering [10–13], and the use of neural networks [14–17], both for supervised and unsupervised problems.

Most of these classification efforts concentrate on the interpretation of individual CPT data, while classification of a regional-scale CPT dataset is generally limited to the use of classical empirical classification charts [18–21], although there are a few recent exceptions [22]. Moreover, geostatistical interpretations or lithostratigraphic mapping of site-specific SBTs at a regional scale (at least several tens of km^2^) have not received much attention. Studies of the spatial variability of CPT data are mainly concerned with geostatistical analysis of the vertical direction [23–25], two-dimensional interpolation of continuous parameters derived from each single CPT test [26, 27], or three-dimensional variography of the raw CPT data or derived continuous parameters such as grain size distribution parameters [28–31]. Little or no work has been done to date on quantification of spatial variability of the SBT classes themselves, which would provide unique insights about different sedimentary facies or lithostratigraphic units, especially at larger spatial scales.

The above-mentioned classification methods are so-called unsupervised heuristic clustering methods (hierarchical and *k*-means), whose main limitations are determined by their underlying probability models [32]. The standard *k*-means clustering algorithm, for instance, yields equal-volume hyperspherical clusters which might lead to unnecessary partitioning of the true classes within the data. Moreover, the standard *k*-means algorithm requires that the number of clusters is provided as input, which often is an arbitrary choice. Extensions of the *k*-means algorithm were developed to overcome this problem. The *x*-means approach [33–35] is one solution, where a more efficient algorithm is combined with the use of the Bayesian Information Criterion (BIC) to provide both the number of clusters and their parameters. The model-based clustering approach of Fraley and Raftery [29, 36] goes further by using mixture models with an expectation-maximization (EM) algorithm, generalized to incorporating different underlying probability models.

We here compare the *x*-means and more traditional methods from literature to the model-based clustering approach. To facilitate robust lithostratigraphic mapping using discrete SBTs, a novel methodology is presented for the automated lithostratigraphic mapping of sedimentary units at a scale of several tens of km^2^, making use of a site-specific SBT classification. The automated mapping approach is compared with results from the more traditional manual approach using SBT classification diagrams from literature [17–19].

The clustering algorithms and lithostratigraphic mapping are applied to the CPT dataset of a ~60 km^2^ groundwater basin with predominantly unconsolidated sandy sediments in northern Belgium. The results are assessed with available borehole data [37], lithostratigraphic mapping using the traditional manual approach [16–19], and the resulting spatial indicator variability.

## Methodology

### Basic CPT parameters

Typical raw CPT data includes the cone tip resistance, *q*_c_, and the sleeve friction, *f*_s_ [1] (an overview of all symbols is provided in Table 1). Analysis of raw CPT data (*f*_s_ and *q*_c_) has traditionally been done by derivation of parameters like friction ratio (*R*_*f*_), normalized cone tip resistance (*Q*_*t*_), and normalized friction ratio (*F*_*r*_) and their subsequent use in existing charts or classifications. The friction ratio (*R*_*f*_) represents the ratio between *f*_s_ and *q*_c_:

$${\text{R}}_{\text{f}}={\text{f}}_{\text{s}}/{\text{q}}_{\text{c}}\times 100\text{\%}$$ (1)

A correction can be applied for the pore pressure in case of CPTu measurements, which results in the corrected cone tip resistance, *q*_t_

$${\text{q}}_{\text{t}}={\text{q}}_{\text{c}}+\text{u}\left(1-\text{a}\right)$$ (2)

With *u* the pore pressure and *a* the net area ratio determined by the characteristics of the used cone. Stress-normalized equivalents of the variables *q*_t_ and *R*_f_ should be used to account for the in-situ vertical stresses: the normalized cone tip resistance, *Q*_*t*_

$${\text{Q}}_{\text{t}}=\left({\text{q}}_{\text{t}}-\sigma_{\text{v}0}\right)/\sigma_{\text{v}0}^{\text{'}}$$ (3)

and the normalized friction ratio, *F*_*r*_

$${\text{F}}_{\text{r}}={\text{f}}_{\text{s}}/\left({\text{q}}_{\text{t}}-\sigma_{\text{v}0}\right)\times 100\text{\%}$$ (4)

with σ_v0_ the total overburden pressure, and $\sigma_{\text{v0}}^{\prime }$ the effective vertical stress. Jefferies and Davies [38] introduced the SBT index *I*_c_ to represent the radius of the concentric circles in the classification diagram of Robertson [5]. We use the Robertson and Wride [39] expression for *I*_c_:

$${\text{I}}_{\text{c}}={\left({\left(3.47-{\text{log Q}}_{\text{t}}\right)}^{2}+{\left({\text{log F}}_{\text{r}}+1.22\right)}^{2}\right)}^{0.5}$$ (5)

where *Q*_t_ and *F*_r_ are as defined in Eqs 3 and 4. The *I*_c_ variable captures only the soil type from the raw CPT data, and carries little or no information on the in-situ soil state (consolidation, cementation, or sensitivity, *i*.*e*. ratio of the strength of the soil in the undisturbed state to that of the soil in the remolded state). In contrast, the 2D classification charts [4, 5] do include such additional soil state information.

**Table 1:** List of symbols.

Symbol	Explanation
CPT	Cone penetration test
SBT	Soil behaviour type
q_c_; q_t_; Q_t_	Measured, corrected and normalized cone tip resistance (MPa)
f_s_	Sleeve Friction (MPa)
R_f_; F_r_	Measured and normalized friction ratio (%)
U	Pore water pressure (MPa)
a	Net area ratio (dimensionless)
σ_v0_; $\sigma_{\text{v0}}^{\prime }$	Total and effective in-situ vertical stress (MPa)
I_c_	Soil behaviour type index
z_strat_	Stratigraphic depth (elevation in meter above top aquitard)
z_ref_	Reference elevation for z_strat_, corresponding to the top of the aquitard
z_masl_	Elevation in meter above sea level
z_mbgl_	Depth in meter below ground level

### Field site

**Fig 1:**
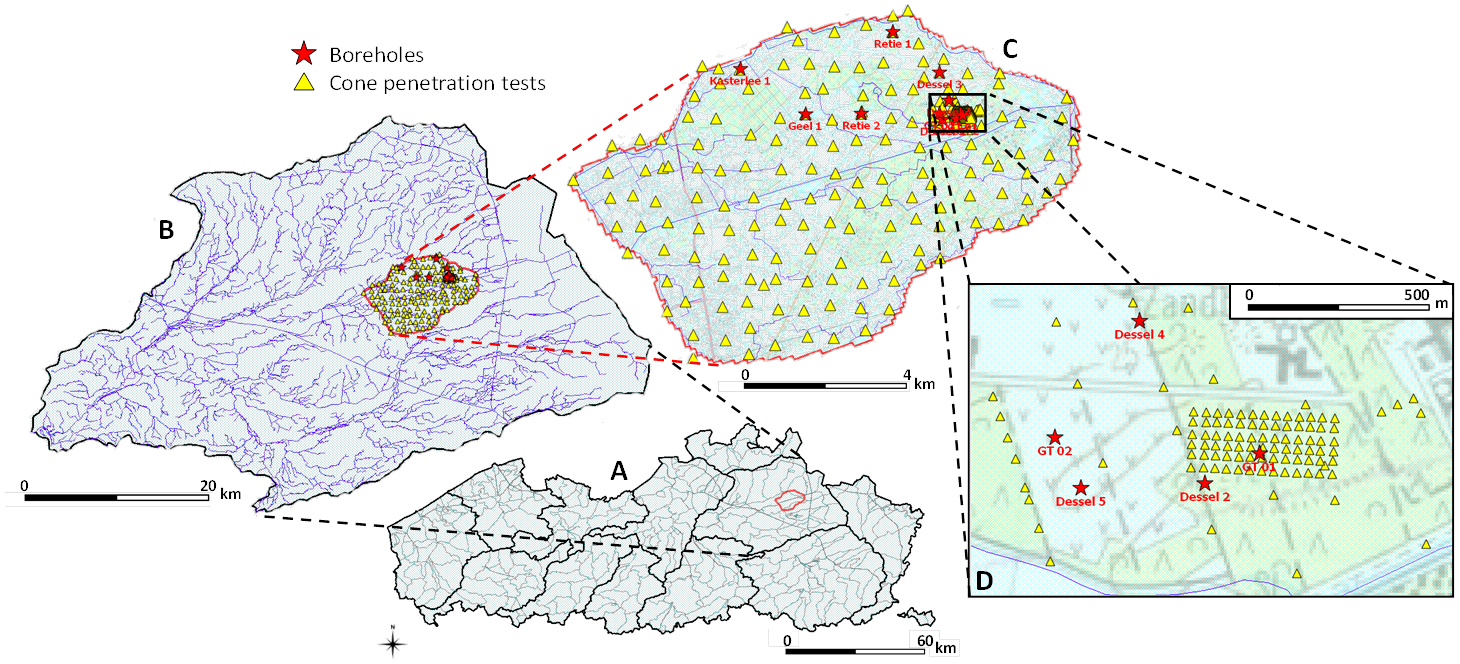
Location of the study area within Flanders, Belgium (A) and the Nete basin (B), and location of CPTs and cored boreholes for the coarse (C) and fine sampling grid (D).

A detailed hydrogeological characterization of Quaternary and Neogene sediments, commissioned by ONDRAF/NIRAS (the Belgian National Agency for Radioactive Waste and enriched Fissile Material), reaching depths of up to 40 to 50 m has been carried out in 2008–2010 within the Nete basin, north Belgium (Fig 1A and 1B) [34]. A large amount of hydrogeological information has been collected in an area of about 60 km^2^ (and permission was granted by ONDRAF/NIRAS for its use in this work), including nearly 400 m of continuous borehole cores, wireline logs from boreholes including natural gamma radiation and electrical resistivity, about 200 CPTs on a quasi-regular 600x600 m sampling grid and about 90 on a finer 30x30 m grid, and various hydrogeological measurements on undisturbed cores (Fig 1C and 1D) [34].

Since most of the data originate from standard CPTs while trial corrections for the small number of CPTu (with pore pressure registration) tests proved to be insignificant (mainly due to the shallow depths involved), the corrected cone tip resistance formulation using pore water pressure (Eq 2) is not applied in this study. The cone area for all CPT tests was 1500 mm^2^. The tests reached depths between 15 and 42 m, with 60% of tests over 30 m deep.

**Fig 2:**
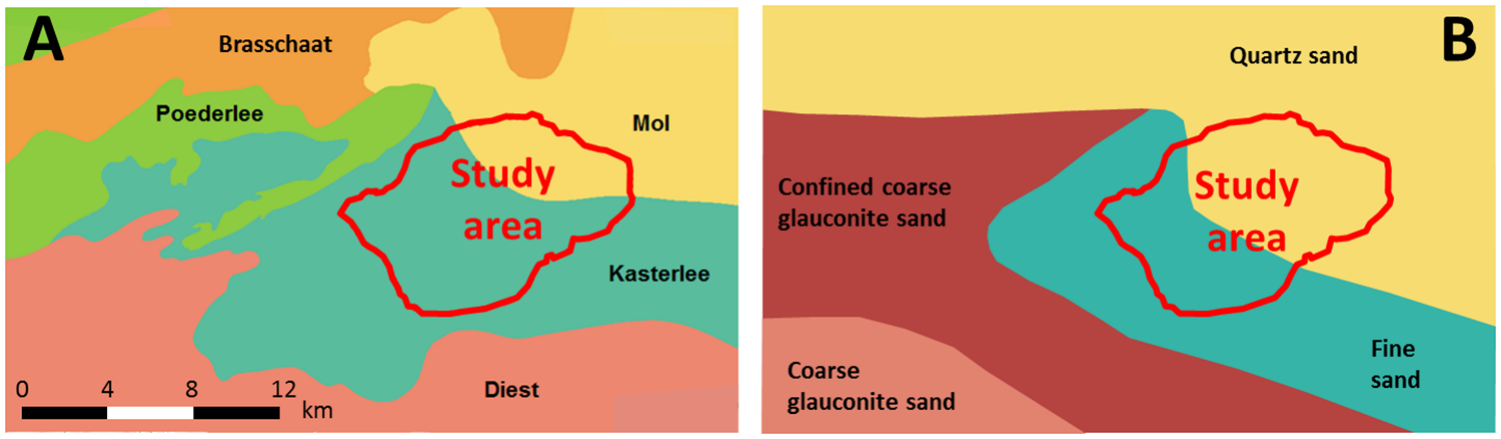
Geological (A) and hydrogeological map (B) of the study area and its surroundings, respectively based on DOV [47] and Gulinck [48].

Several boreholes were drilled in the study area (see Figs 1 and 2), of which seven were fully cored in the upper 40 to 50 meter. After the drilling operations the continuous cores were used for stratigraphic analysis and sampling; a range of sediment properties were determined nearly every two metres along the cores, including saturated hydraulic conductivity, porosity, bulk density, grain size, glauconite content and cation exchange capacity [34].

The first set of CPT tests on the 600x600 m sampling grid was aimed at mapping the geometry and thickness of an aquitard with a maximum depth of 30–40 m in the study area. A second set of CPTs was performed on the 30x30 m sampling grid to define small-scale variability in stratigraphy, and a third set of CPTs was obtained at short distances (between 1.5 and 5 m) from the cored boreholes. The latter allows comparison between the CPT data and SBT classifications to the sediment properties obtained from the borehole cores and wireline logs.

**Fig 3:**
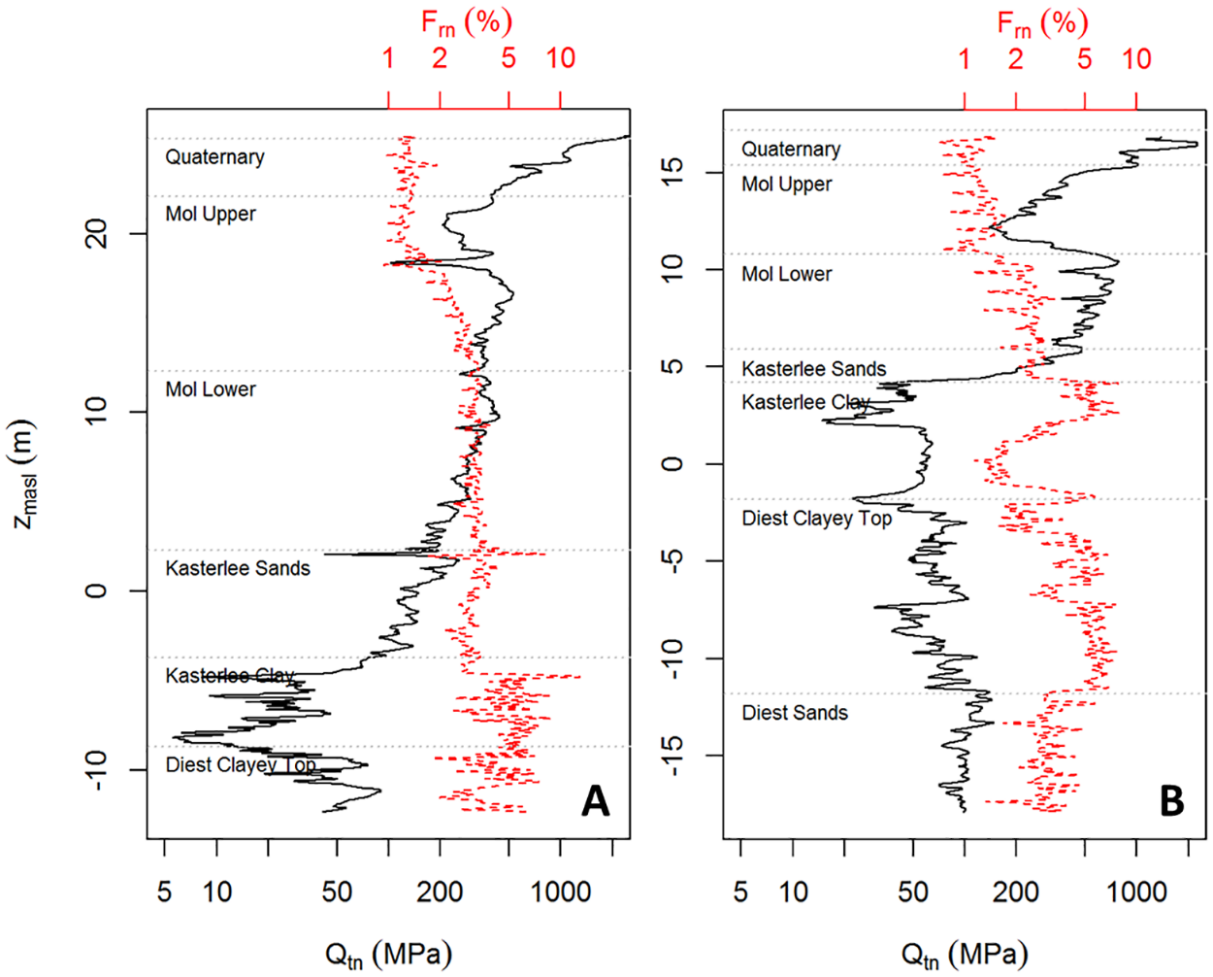
Two example CPT logs displaying normalized cone tip resistance (*Q_t_*) and normalized friction ratio (*F_r_*). Their location is indicated in Fig 4. Stratigraphy is based on nearby (< 10 m) boreholes “Dessel-2” (A) and “Kasterlee-1” (B) (see Fig 1).

**Fig 4:**
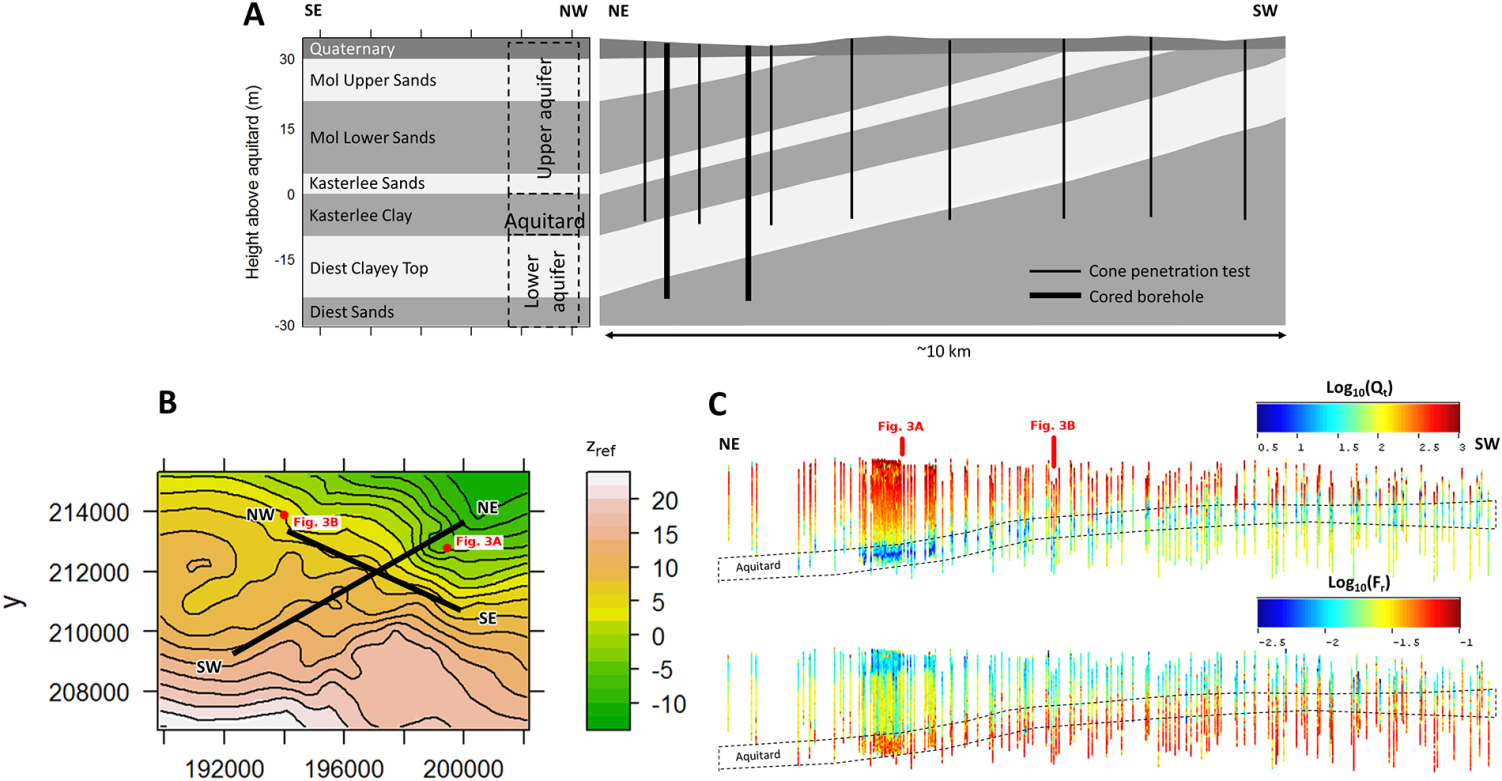
A) Conceptual lithostratigraphic profiles through the study area. B) Top view of the location of the profiles in A and C with respect to the geometry of the top of the aquitard. C) Sideview of the CPT data (40x height exaggeration with panel dimension ~10 km x 40 m) projected orthogonally onto the NE-SW dipping plane (which corresponds to the NE-SW conceptual profile in A), with logarithmic normalized cone tip resistance (*Q*_t_) and friction ratio (*F*_r_).

The local lithostratigraphic succession consists of, from top to bottom, various Quaternary deposits, the Mol Upper Sands, the Mol Lower Sands, the Kasterlee Sands, the Kasterlee Clay, the Diest Clayey Top, and the Diest Sands (Figs 2A, 3, and 4A).

The Quaternary sediments mainly consist of different phases of eolian deposits and to a lesser degree alluvial deposits from the Nete rivers [40, 41].

The Pliocene Mol Formation consists of white, coarse and medium fine sands. It is sometimes lignitic and can contain some lenses of micaceous clay [42]; only the latter has been reported in the current study area. The bottom part of the ~20 m thick formation has low levels of glauconite (< 2%) [34]. In the current study area, the Pliocene Mol Formation is divided into the Mol Upper and Mol Lower Sands [34]. The latter are very well sorted, finer and darker in colour, while the former are moderately to well sorted medium sands with a basal gravel layer. Because of the high siliceous content of this sand (99% SiO_2_) [43], it is being mined for various industrial uses [44].

The Miocene Kasterlee Formation consists of a relatively homogeneous fine, micaceous, slightly glauconitic, sandy upper part [39], and a very heterogeneous alternation of clay lenses (clay contents of up to 40%) and sand banks in the lower 5–7 m [34, 45, 38]. The more homogeneous upper part (~ 1.5–6 m thick) is referred to as Kasterlee Sands while the heterogeneous clay-rich lower part is named Kasterlee Clay.

The Diest Formation consists of grey-green to brownish, mostly coarse and locally clayey glauconiferous sand, often with sandstone layers [39]. In the current study area, a distinction is made between the clayey upper part of the formation (~ 10 m thick), the Diest Clayey Top, and the Diest Sands below (up to 80 m thick). Glauconite content in the Diest Sands can be as high as 50 weight percent [34].

The geological map including these formations is displayed in Fig 2A. The Poederlee [39] and Brasschaat [46] Formations are lateral equivalents of the Mol Formation (see the hydrogeological map Fig 2B), and overlie the Kasterlee Formation (or its lateral equivalents) northeast of the study area. As these lateral transitions are probably more gradual than suggested by the geological map, we can also expect some trends in the sediment properties within our study area. The hydrogeological map also clearly indicates the presence of the Kasterlee Clay aquitard at shallow depth to the west and south of our study area, with part of the coarse glauconite sand (Diest Formation) being indicated as confined. The aquitard becomes deeper moving eastward and under the study area its top typically occurs between 5 and 40 m below surface.

Two example CPT logs showing normalized cone tip resistance (*Q*_*t*_) and normalized friction ratio (*F*_r_) are displayed in Fig 3. The most remarkable features are the high *Q*_*t*_ values for the Quaternary sands, and the low but highly variable *Q*_*t*_ and high *F*_*r*_ values for the Kasterlee Clay, especially in profile A. Furthermore, the Diest Clayey top is more similar to the Kasterlee Clay than the underlying Diest Sands. The latter sandy layers have considerably different *Q*_t_ and high *F*_*r*_ values compared to any of the sands, i.e. Mol or Kasterlee Sands.

While the Quaternary deposits unconformably overlie the Neogene formations, the latter are all inclined, and dipping towards the North-East as shown by the conceptual profiles in Fig 4A and the different vertical positions of the layers in Fig 3 (A is east of B). A sideview of the entire CPT data set, projected orthogonally onto the NE-SW dipping plane, is shown in Fig 4B. This overview clearly shows the differences in both *Q*_*t*_ and *F*_*r*_ between the upper aquifer (from Quaternary sands to Kasterlee Sands) and lower aquifer sediments (from Diest Clayey Top down), separated by the Kasterlee Clay aquitard which has the overall lowest *Q*_*t*_ values (thin blue layer in Fig 4B). On the basis of the *F*_r_ values alone the most clay-rich layers, i.e. Kasterlee Clay and the underlying Diest Clayey Top, cannot be distinguished easily. Conversely, visual separation of Quaternary sands and Mol Upper Sands on the basis of *F*_r_ values becomes more apparent in the NE section of the data panel where the lowest recorded *F*_r_ values occur.

Previously Schiltz [17, 18] manually delineated all the lithostratigraphic boundaries except those for Quaternary—Mol Upper and Mol Lower—Kasterlee Sands horizons, using this CPT data set and the measured (*q*_c_ and *f*_s_) and derived (*R*_f_, see Eq 1) parameters combined with the SBT classification of Robertson *et al*. [4]. Continuous 2D maps of these derived lithostratigraphic boundaries were obtained by universal kriging [16]. Our current analysis extends this earlier work by developing a novel automated classification method for identification of lithostratigraphic boundaries.

The top of the Kasterlee Clay aquitard, referred-to as *z*_ref_, is the most pronounced and most easy to discern lithostratigraphic boundary using CPT or other data. It depends only on the *x* and *y* coordinates, and is used in this paper to derive the stratigraphic depth *z*_strat_, which represents the position of a given point with coordinates *x*, *y* and *z*_masl_ (meter above sea level) in the stratigraphic column. To obtain *z*_strat_, the value of *z*_ref_ is subtracted from all absolute height values, z_masl_, such that z_strat_ (*x*, *y*, *z*_masl_) = z_masl_−z_ref_ (*x*, *y*). The reference horizon equals the surface defined by z_strat_ = 0. The use of this depth parameter is tested within the site-specific clustering to investigate the effects on the match between SBT classes and the true lithostratigraphy.

### Data classification

#### Soil behaviour type (SBT) classification

**Fig 5:**
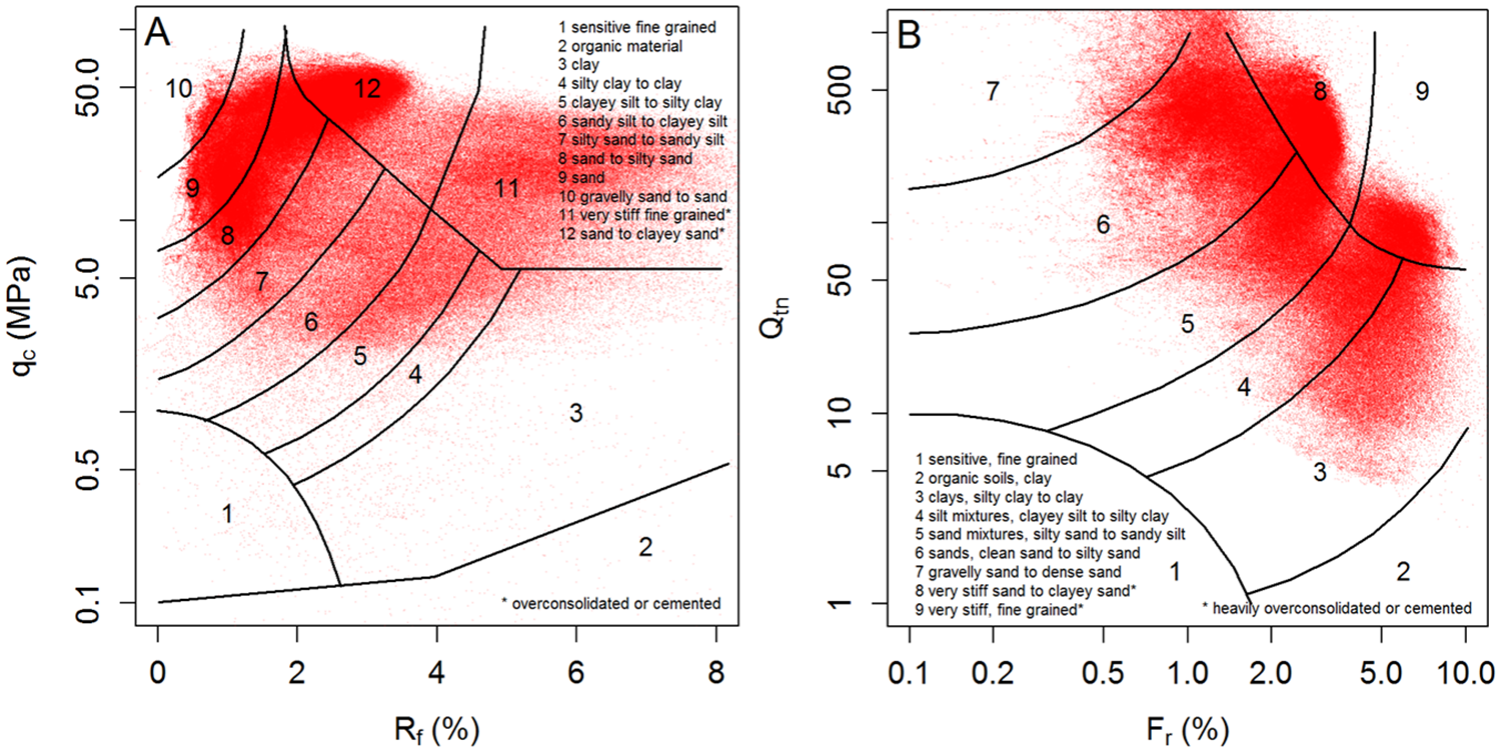
SBT classification charts of Robertson et al. [4] (A) and Robertson [5] (B). Data (~ 480,000 data points) from this study are shown as red dots.

Among the existing SBT classification methods in literature, those of Robertson *et al*. [4] and Robertson [5] are probably the most frequently used (Fig 5). Only the latter method uses the normalized CPT variables to account for the increasing overburden pressure with depth. Moreover, the number of SBTs is also different, with more classes in the silt-sand range for the first classification. Even though updates were recently provided for these classification charts [49, 50], the original 1986 version is used in this paper for comparison with the other approaches since its use is more widespread.

**Fig 6:**
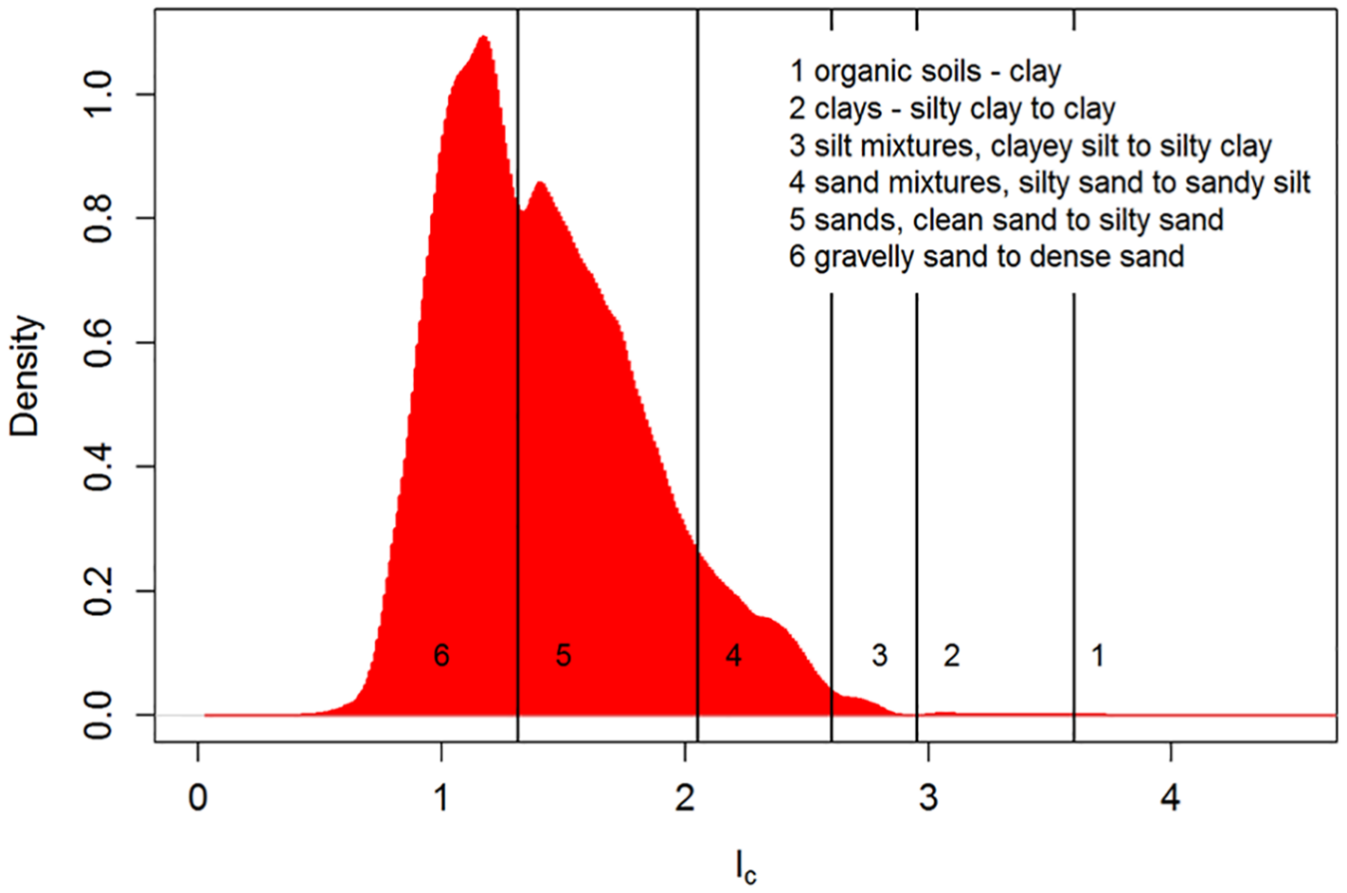
SBTs density plot based on the SBT index *I*_*c*_. Data are from this case study with ~ 480,000 observations.

Since such diagrams use *a priori* defined classes and are thus not site-specific in any way, these classifications are purely descriptive and probably lack the means of finding the true typology of the data. This is illustrated in Fig 5 where the data are plotted onto the diagrams. Several clusters of data points clearly intersect different regions of the diagram, and would not be classified as one consistent (though heterogeneous) SBT type, which complicates interpretation of stratigraphy or facies. Therefore, a site-specific classification might provide a better solution. Another approach is the use of ranges of the SBT index *I*_c_ (calculated with Eq 5) to define SBT classes like the one presented in Table 2 and Fig 6 [51]. These results suffer from the same limitations as the SBT classification diagrams, although now only in one dimension (*i*.*e*. one variable only: *I*_c_).

**Table 2:** SBT classification based on SBT index (*I*_c_) ranges [47].

SBT nr.	SBT index (*I*_*c*_) range	Lithology
1	> 3.60	Organic soils—clay
2	2.95–3.60	Clays—silty clay to clay
3	2.60–2.95	Silt mixtures—clayey silt to silty clay
4	2.05–2.60	Sand mixtures—silty sand to sandy silt
5	1.31–2.05	Sands—clean sand to silty sand
6	< 1.31	Gravelly sand to dense sand

Even though a classification system may effectively separate data into distinctly different clusters, as exemplified in Fig 5B for class 4 and 5 or in Fig 6 for class 5 and 6, overlap between adjacent classes complicates the analysis. To resolve this, a probabilistic clustering approach (*e*.*g*. model-based clustering) is proposed, where data points are not assigned to a single class, but rather are given probabilities of belonging to all different classes.

#### *k*- and *x*-means clustering

The *k*-means clustering approach is one of the most frequently used unsupervised clustering techniques, mostly due to the straightforward implementation of the standard algorithm, and its limited computational time requirements compared to more complicated methods. Standard *k*-means clustering minimizes within-cluster distances while maximizing between-cluster distances, through minimizing the objective function

$$\;\sum_{i=1}^{k}\sum_{j=1}^{n}||x_{j}^{i}-\mu_{i}|{|}^{2}$$ (6)

with *k* the number of clusters, *n* the number of data points within each cluster, $x_{\text{j}}^{\text{i}}$ data point j of cluster i, and μ_i_ the centre of cluster i. Minimization of the objective function is typically achieved through the following procedure: 1) choose the number of clusters *k*, 2) initialize *k* cluster centres randomly within the multivariate data space, 3) classify all data points to the closest cluster (minimum distance to cluster centre), 4) recalculate the cluster centres by taking the average or centroid of all data points, and 5) repeat step 3 and 4 and iterate until convergence is reached (classification does not change). Since the algorithm is heuristic, many initializations may be required to assure finding the global optimum. In practice, however, a small number of initializations is usually sufficient [52]. Several versions including more efficient adaptations of this algorithm exist [53–56].

The initial *x*-means extension of the *k*-means algorithm [30] uses splitting of the clusters after each *k*-means iteration to better fit the data according to the Bayesian Information Criterion (BIC) which approximates the hard to evaluate integrated likelihood

$$2\text{ log p}\left(\text{X }|\text{ M}\right)\approx 2\text{ log p}(\text{X }|\hat{\theta },\text{M})-v\text{ log}\left(m\right)=\text{BIC}$$ (7)

where *X* represents the dataset, $\hat{\theta }$ the maximum likelihood estimate of the parameters *θ*, *M* the model, *v* the number of parameters and *m* the number of data points.

When the BIC does not improve any further by splitting clusters, the optimal number of clusters is reached. The magnitude of the variance and covariance around the cluster centres are also considered for evaluation of the progressive division using the BIC [31]. More recently, a cluster merging operation was added to the algorithm, to prevent unsuitable division of clusters due to the splitting order [32]. We use the implementation of this algorithm in the R language (available from http://www.rd.dnc.ac.jp/~tunenori/xmeans_e.html). In this paper, we only apply *x*-means clustering, as it is superior to the traditional *k*-means approach, and represents the most frequently used deterministic unsupervised classification algorithm.

The optimization is based on a maximum number of iterations of 1000, which was tested prior to the final analysis to ensure convergence and identification of the global optimum; the initial number of clusters is set to two. To avoid effects due to variables exhibiting different units and/or variances, data standardization is performed prior to the clustering. For a dataset of ~300000 observations, and the variables considered in this paper, the algorithm execution time was between ~10 and ~40 seconds on a 2.4 GHz CPU, and resulted in two to four classes for the different approaches considered.

#### Model-based clustering

Model-based clustering [29] consists of fitting a mixture of *k* multivariate normal densities to a multivariate dataset, where the *i*-th multivariate normal density *Φ*_i_, parameterized by its mean *μ*_*i*_ and covariance matrix *Σ*_i_, is represented by

$$\Phi_{\text{i}}\left(\text{x }|\mu_{\text{i }},\Sigma_{\text{i}}\right)=\frac{\text{exp}\left\{-\frac{1}{2}{\left(\text{x}-\mu_{\text{i}}\right)}^{\text{T}}\Sigma_{\text{i}}^{-1}\left(\text{x}-\mu_{\text{i}}\right)\right\}}{\sqrt{\text{det}\left(2{\text{πΣ}}_{\text{i}}\right)}}$$ (8)

where *x* = (*x*_1_,…,*x*_N_) for an N-dimensional dataset. Expectation maximization (EM) can be used to obtain the best fit, given the number of clusters *k*. The EM algorithm iterates between two steps: 1) the E step, in which the probability of each observation belonging to each cluster using the current parameter estimates (means and variances) is computed, and 2) the M step, in which model parameters are estimated using the current group membership probabilities. For details on the implementation of this algorithm for mixture modelling the reader is referred to Fraley and Raftery [29]. The software package MCLUST [33, 57] is used here. MCLUST can perform model-based clustering for all numbers of classes specified and with a number of different covariance matrix parameterizations. The most simple case is the equal volume spherical model (covariance matrix Σ_i_ = *λI*, with *I* the identity matrix and *λ* the common variance), which is similar to the underlying model of *k*-means clustering. The most complicated case is the unconstrained model ($\Sigma_{\text{i}}=\lambda_{\text{i}}D_{\text{i}}A_{\text{i}}D_{\text{i}}^{\text{T}}$, with *D* an orthogonal matrix that specifies the orientation and *A* a diagonal matrix that specifies the shape), which allows all clusters to have a different shape, volume and orientation. Hierarchical clustering is used for the initialization of the EM algorithm, and the best model is again selected according to the BIC.

The disadvantage of the MCLUST algorithm for the unconstrained model, which is applied here, is the increase in computational time. For the different cases run in this paper, execution times were between ~10 seconds and ~10 minutes, mainly depending on the number of classes obtained (between 4 and 19 in this case). Theoretically, standardization of the variables is not necessary due to the algorithm flexibility, but to avoid problems and to speed up the convergence, standardization is applied before clustering, as in the *k*-means approach. Given the high sensitivity of model-based clustering to data density, all subsets of the data which were subjected to the algorithms (see Table 3) were sampled from a uniform distribution of the stratigraphic depth, z_strat_. Such uniform distribution should avoid creating artificial clusters due to a different sampling density for different positions in the lithostratigraphic column.

**Table 3:** Number of SBT classes for the different classification approaches.

Method	*k*
Literature *I*_c_ [47]	6
Literature *q*_c_−*R*_f_ [4]	12
Literature *Q*_t_−*F*_r_ [5]	9
*x*-means: *I*_c_	2
*x*-means: *I*_c_−*z*_strat_	2
*x*-means: *Q*_t_−*F*_r_	3
*x*-means: *Q*_t_−*F*_r_−*z*_strat_	4
MCLUST: *I*_c_	4
MCLUST: *I*_c_−*z*_strat_	12
MCLUST: *Q*_t_−*F*_r_	14
MCLUST: *Q*_t_−*F*_r_−*z*_strat_	19

Both *x*-means and model-based clustering algorithms are applied to four combinations of CPT variables (codes used in subsequent discussions): only *I*_c_ (*I*_c_); *I*_c_ with stratigraphic depth z_strat_ (*I*c−*z*_strat_); *Q*_t_ with *F*_r_ (*Q*_t_−*F*_r_); and *Q*_t_, *F*_r_, and *z*_strat_ (*Q*_t_−*F*_r_−*z*_strat_). *I*_c_ was selected because it merges most of the available information into a single variable. Furthermore, *Q*_t_ and *F*_r_ were selected because of their proven use in the classical SBT classification charts. The reason for including *z*_strat_ is that it represents the main direction of heterogeneity within the study area. Moreover, it allows for the detection of different lithostratigraphic units that share the same properties in terms of *I*_c_ or *Q*_t_ and *F*_r_, but that are in a different position in the stratigraphic column.

### Regional lithostratigraphic mapping

To test the usefulness of site-specific SBT classification in mapping the occurrence of certain lithostratigraphic units or boundaries, we propose the following methodology for automated classification and delineation of such units or boundaries: 1) perform clustering of a selection of CPT parameters with a target number of 2 classes for the entire dataset, 2) only retain CPTs with a given minimum number of data points in both classes to ensure the boundary between the two classes is actually penetrated by the CPT, and 3) calculate the crossing points of the normalized density estimates for both classes along the *z* axis. When mapping a lithological unit instead of a lithostratigraphic boundary, the class with maximum density can be assigned to the respective data points.

**Fig 7:**
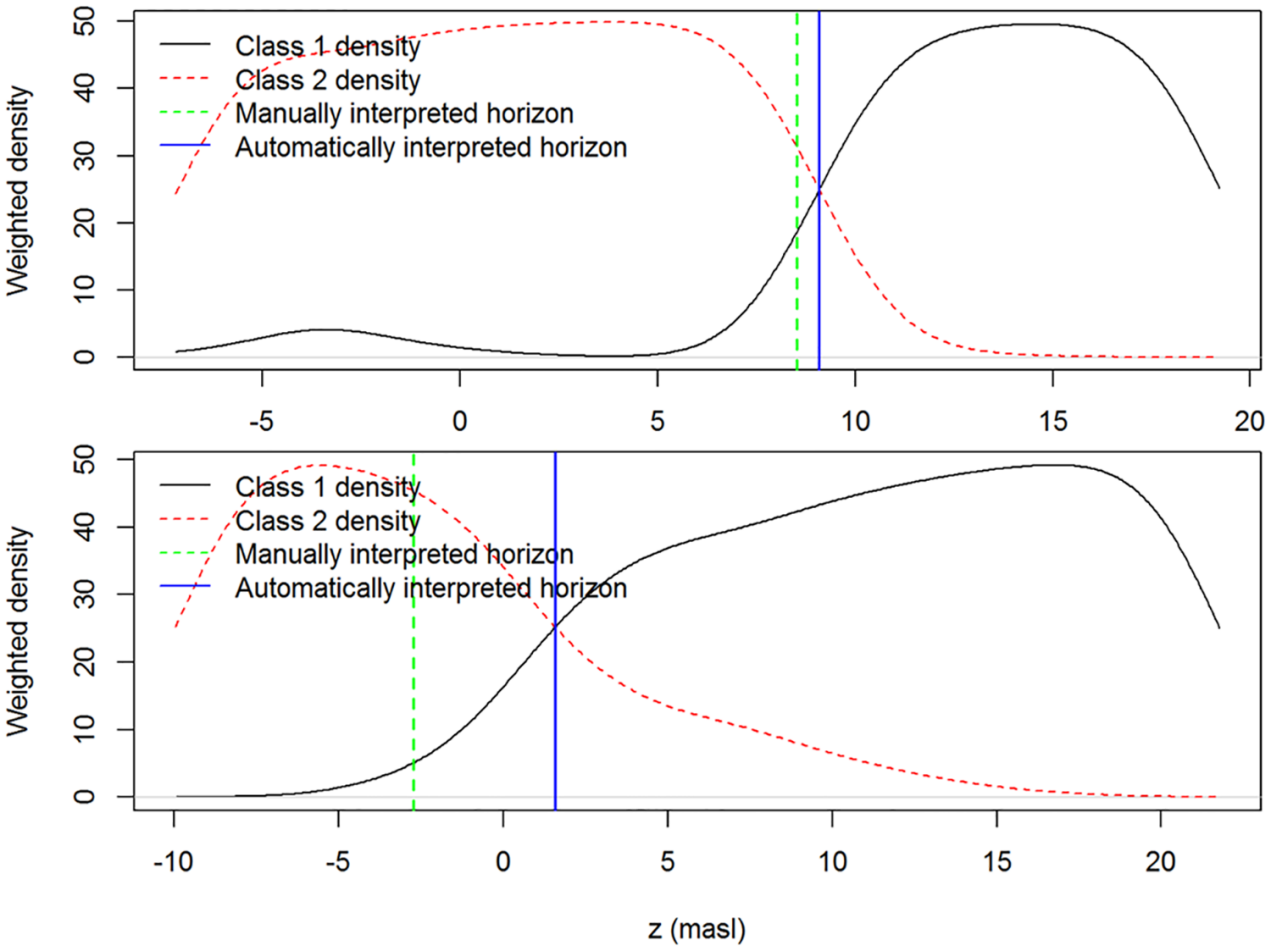
Examples of an automatically selected horizon mapped by using model-based clustering and the kernel density estimates of the *z* coordinates of the two contrasting classes.

We apply this automated classification approach to the top surface of the Kasterlee Clay aquitard, which is also fairly easy to map manually using traditional SBT classification diagrams as indicators for lithology. The manual mapping of the surface was performed by Schiltz [17, 18] and reported by Wouters and Schiltz [19], and is used as a reference in this study. For the site-specific clustering, we use both the *x*-means and MCLUST algorithms with *I*_c_ as the CPT variable. For each CPT log the minimum number of data points in each SBT class was put to 150, which corresponds to ~3 m out of a continuous CPT log. An example of such normalized density estimates and the selected horizon is shown in Fig 7.

To create 2D maps of the top surface of the aquitard we used universal kriging [58] with a linear trend model in function of the *x* and *y* coordinates.

### Visualization of class properties

#### Multivariate statistics

For each of the different classifications used in this work, we use biplots to visualize the relationship between the obtained SBT classes and sediment properties independently obtained from the cored boreholes (*i*.*e*. only a very limited part of the CPT dataset). A biplot is an exploratory graph which displays information on both samples and variables of a data matrix [59]. We use the first two principal components of the sediment property dataset for the axes of the biplot, and project the variables as vectors to this plane using the principal component biplot described by Gabriel [60]. For visualising the different SBT classes, we plot all data samples together with the cluster centres *μ*_i_. This provides an assessment of the potential relationship between the different SBT classes and the sediment properties obtained from the borehole cores, and gives an idea on the usefulness of the clustering method used. Cluster centres showing the same multivariate properties (plotted near each other in the biplot), therefore might indicate an arbitrary division of a single SBT class, while an even spread of cluster centres indicates that the obtained SBT classes better reflect the true typology of the subsurface sediments. For a more complete description on the construction and interpretation of biplots, the reader is referred to Gower and Hand [55].

#### Spatial distribution

To investigate the SBT spatial distribution obtained from the different SBT classification methods, we used the following approach: 1) determine the marginal distributions of all SBT classes for each recorded meter of the vertical stratigraphic succession, 2) convert the SBT classifications to *k* SBT indicators, 3) perform indicator variography using the gstat package [61] and fitted spherical variogram models using a least squares approach with minimum and maximum semi-variance as initial nugget and total sill values and 3 and 1000 m for the initial vertical and horizontal ranges, and 4) analyse the regional distribution of classes within the entire 3D dataset by using an orthogonal projection of a sideview of the data, perpendicular to the strata dip.

## Results and discussion

### Number of SBT classes

The three literature SBT classifications (*q*_c_−*R*_f_, *Q*_t_−*F*_r_, and *I*_c_) were applied to all CPT data and have respectively 12, 9, and 6 classes. The *x*-means and MCLUST algorithms were applied to the four CPT-derived datasets (*I*_c_, *I*_c_−*z*_strat_, *Q*_t_−*F*_r_, and *Q*_t_−*F*_r_−*z*_strat_) resulting in different numbers of SBT classes for the different datasets (Table 3). Because the three literature SBT classifications are not site-specific, several of the classes are not or hardly represented within the dataset.

The site-specific classifications resulting from the *x*-means and MCLUST algorithms yield contrasting results. The *x*-means method yields a smaller number of SBT classes ranging from 2 to 4 classes depending on the used dataset. For the MCLUST algorithm, between 4 and 19 classes were derived through optimization, depending on the dataset used.

The *x*-means approach yielded a robust SBT classification with only a few SBTs. However, a too small number of SBT classes might fail to provide the required level of detail to discriminate between different lithostratigraphic units. The MCLUST algorithm provides at most 19 SBTs, which seems too many to provide a comprehensive overview of the dataset. Moreover, such a high number was not expected based on the borehole and lithostratigraphic data.

### Multivariate characteristics

**Fig 8:**
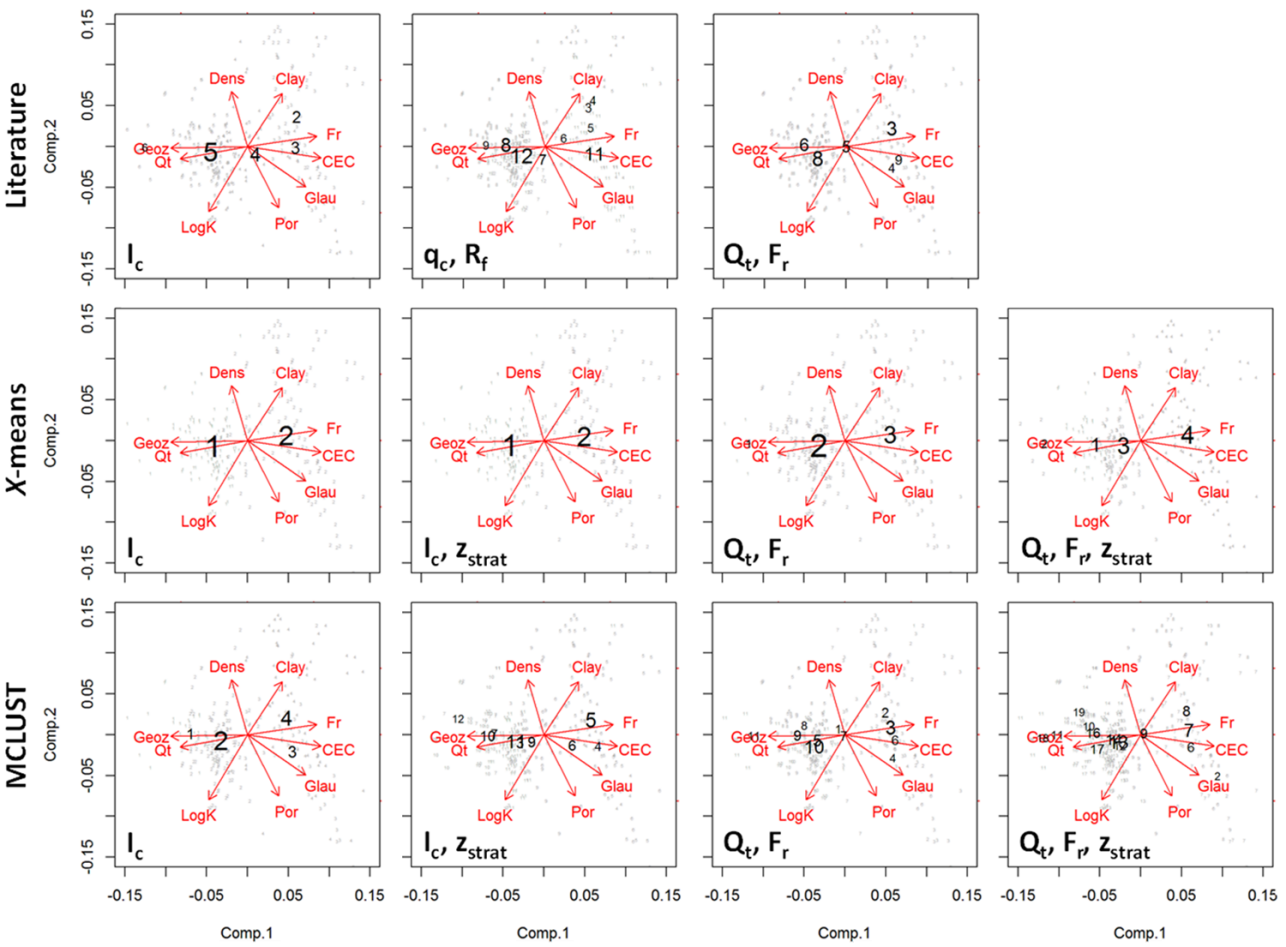
Biplots for all 11 SBT classifications. The first two principal components (Comp.1, Comp.2) of the nine sediment properties (“Geoz” = z_strat_, “Por” = porosity, “Dens” = bulk density, “LogK” = logarithmic hydraulic conductivity, “Clay” = clay content, “Glau” = glauconite content, and “CEC” = cation exchange capacity). The SBT data are represented as individual data points and cluster centres (black numbers). The *x*- and *y*-coordinates of these points are multiplied by 3.5 to illustrate more clearly the relationship with the sediment properties. The size of the numbers is proportional to the amount of data points in the cluster.

The multivariate sediment characteristics of the eleven SBT classifications from Table 3 are shown in the biplots in Fig 8. The size of the SBT class numbers is proportional to the number of data points within a given SBT class. To maximize the information gain from the borehole core dataset for assessing the clustering results, missing sediment property data at certain depths were completed with linear estimates, using the other properties as predictors and the complete data entries to derive the linear model parameters. Based on all classifications, *Q*_t_ correlates positively with *z*_strat_, (arrows pointing in the same direction) and negatively with *F*_r_, and cation exchange capacity, and glauconite content (arrows pointing in opposite direction). Log-transformed hydraulic conductivity is slightly positively correlated to *Q*_t_, and not unexpectedly correlates negatively with clay content. Porosity is negatively and bulk density positively correlated to clay content; there is hardly any correlation with *Q*_t_ or *F*_r_.

The *I*_c_ literature classification provides 5 SBTs with clearly distinguishable properties (Fig 8, top row left column). The sixth class, SBT 6, is hardly present in the borehole dataset, while SBT 1 is not present at all. Most of the SBT cluster centres fall around the *Q*_t_ / *z*_strat_−*F*_r_ / CEC direction, whereas SBT 2 clearly deviates from that with a high clay content. The latter indicates SBT 2 may be identified with the aquitard.

The literature *q*_c_−*R*_f_ classification shows a total of nine SBT classes, all more or less aligned with the *Q*_t_ / *z*_strat_−*F*_r_ / CEC direction (Fig 8, top row second column). SBT 3 and 4 (high clay content classes) almost share the same average properties. This indicates these SBT classes cannot be differentiated at the studied site.

The literature SBT classification of *Q*_t_ and *F*_r_ shows six classes with well defined sediment properties; SBT 4 and 9 almost overlap, and supposedly are an indicator for the Diest Clayey Top (high glauconite content and low z_*strat*_ values).

The *x*-means classifications result in only few SBTs: 2 classes for *I*_c_ and *q*_c_−*R*_*f*_; 3 classes for *Q*_*t*_−*F*_*r*_; and 4 classes for *Q*_t_−*F*_*r*_−*z*_*strat*_. All SBT classes are aligned along the *Q*_t_ / *z*_strat_−*F*_r_ / CEC line. These classifications might be robust in the sense that they represent the biggest differences within the dataset, but clearly lack a separate class for the high clay content aquitard, which is the most important feature at this site.

The model-based clustering of *I*_c_ seems to deliver the most robust result (*i*.*e*. clearly representing the biggest differences within the dataset) in which the aquitard is classified separately (SBT 4 shows increased clay content; Fig 8, bottom row, first column). The SBT classes most likely represent the Quaternary and Mol Upper Sands (SBT 1), the Mol Lower and Kasterlee Sands (SBT 2), the Kasterlee Clay aquitard (SBT 4) and the Diest Clayey Top and Diest Sands (SBT 3). Clustering with both *I*_c_ and *z*_strat_ results in a high number of classes, with a few cluster centres in the upper aquifer data (high *z*_strat_ values) that overlap (Fig 8, bottom row, second column).

The model-based clustering of *Q*_t_ and *F*_r_ shows again some overlap of SBT classes (10 and 7, 3, 9 and 11), and detects classes with varying density, porosity, clay content and hydraulic conductivity in the lower part of the lithostratigraphic column (from high density and clay content to high porosity and *K*: SBT 2, 3, 6, and 4; Fig 8, bottom row, third column). Clustering with *Q*_t_, *F*_r_, and *z*_strat_ again leads to a larger amount of classes, which, due to the overlap of the cluster centres, seem not to have very different average properties.

The conclusion of this analysis is that depending on the classification method and used variables, a large range of different classifications can be obtained, with 2 up to 19 SBT classes. The most interesting classification in terms of lithostratigraphic mapping would be the one with the smallest amount of classes possible, while still identifying all lithostratigraphic units. The model-based clustering of the *I*_c_ parameter seems to best correspond with these requirements, although limited variability is detected within the upper aquifer sands. On the other hand, for a detailed classification within lithostratigraphic units (*e*.*g*. for sedimentary facies mapping) a larger number of classes is preferable, with the model-based clustering results providing a data-based alternative to the literature-based arbitrary classification diagrams.

### Spatial distribution of SBT classes

**Fig 9:**
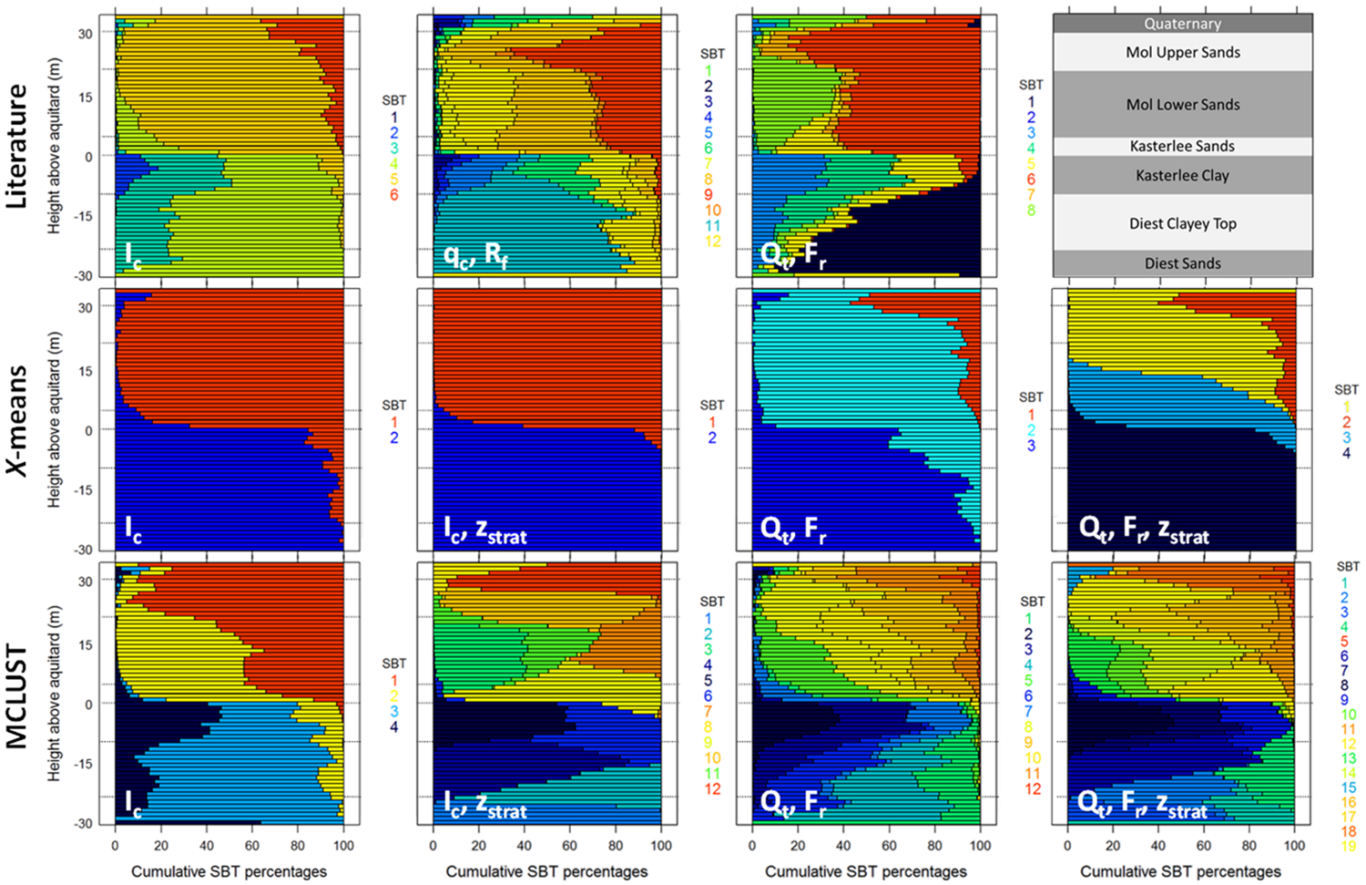
Marginal distributions for SBT classifications along the stratigraphic depth *z*_strat_. Stratigraphic boundaries are overlain based on an average stratigraphic column (top row, last column), and are only indicative.

The marginal distributions of all SBT classes are displayed along the stratigraphic depth *z*_strat_, in Fig 9, together with the approximate location of the lithostratigraphic boundaries (thickness of the different units is not always constant). In displaying the cumulative probability of SBT classes, SBT classes are ordered from left to right according to their geometric average *Q*_t_ (small to large); note the ordering results in different colour codes being used for different classifications. Although a wealth of information is captured in these diagrams, we focus our analysis on the more critical stratigraphic layers such as the Kasterlee Clay aquitard.

The top of the Kasterlee Clay aquitard is clearly discernible for all classifications, typically visible by a large increase in percentage of a single SBT class (mostly the blue or dark blue classes), or the sudden appearance of a new SBT association (*i*.*e*. a group of co-occurring classes, *e*.*g*. the blue, green and yellow classes for the literature *q*_c_−*R*_f_ classification) at a depth of *z*_strat_ = 0. For the *x*-means classifications, the bottom of the aquitard is not identifiable, *i*.*e*. there is no change in any of the SBT classes’ cumulative probability. As a result, the aquitard bottom remains undetected. This is mainly a consequence of the *x*-means algorithm limitations in detecting the classes within the data. There is thus too little detail within these classifications to differentiate between clay-rich layers and glauconite-rich stiff sands. Other classifications (literature *Q*_t_−*F*_r_; MCLUST *I*_c_) do show a decrease of the low *Q*_t_ SBTs, but they remain present within the entire lower aquifer, making delineation of the Diest Clayey Top boundaries difficult. The literature *I*_c_ and *q*_c_−*R*_f_ classifications clearly show one (SBT 2) or more (SBT 2, 3 and 4) SBTs that identify the aquitard. However, based on these classifications, a distinction between the Diest Clayey Top and the Diest Sands remains difficult. The three remaining MCLUST classifications do show SBTs that identify both the aquitard and the Diest Clayey Top layer, with the *I*_c_−z_strat_ classification providing the overall best separation.

Distinction between the four different sandy units in the upper aquifer is hard to make with the literature *I*_c_ and *x*-means *I*_c_ and *I*_c_−*z*_strat_ classifications. The other literature and *x*-means classifications do provide some indication for the Quaternary and Mol Upper Sands, but the Kasterlee Sands remain difficult to distinguish. The same holds for the MCLUST *I*_c_ results, but the other three MCLUST classifications, especially those using *z*_strat_, seem to be more informative. For instance, the *I*_c_−*z*_strat_ classification provides fairly unique cumulative SBT probabilities for each of the sandy layers, *i*.*e*. occurrence of SBT 12 for Quaternary, SBT 10 for Mol Upper, SBT 7 for Mol Lower and SBT 9 for the Kasterlee Sands.

Overall, the literature classifications are useful to provide indications on lithology, and for identifying the aquitard. The *x*-means classifications provide too little detail and are able only to define the top of the aquitard. The higher number of classes in the MCLUST classifications are suited for lithostratigraphic mapping using SBT associations, or single SBTs as indicators for both sandy and clayey lithostratigraphic units.

**Fig 10:**
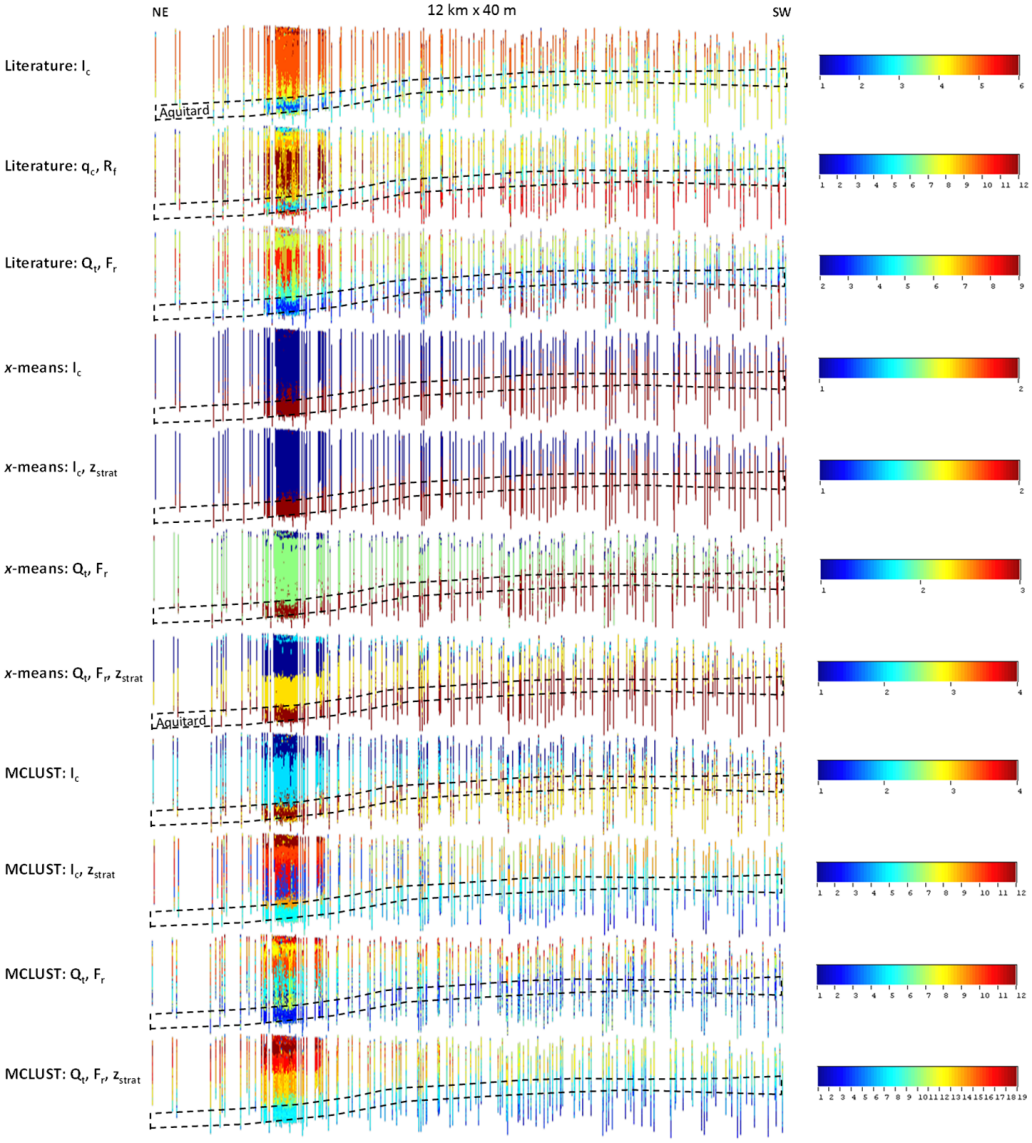
Side views of the CPT dataset (40x height exaggeration; ~10 km x 40 m) projected on a hypothetical plane approximately perpendicular to the layer dip, with colour-coded SBT classes.

The same side view of the entire CPT dataset as in Fig 4B is shown in Fig 10, with the resulting SBT classes instead of the continuous original CPT parameters. The same observations can be made as those from the marginal distributions (Fig 9), although a clear difference now exists between the literature and site-specific classifications. For the former, especially *q*_c_−*R*_f_ and *Q*_t_−*F*_r_, different classes almost randomly alternate at short distances within certain sections of the upper aquifer. This indicates the separation between these classes is purely artificial, and in reality a single SBT exists that covers multiple sections of the respective classification diagrams. The random alternation of SBT classes does not occur with the site-specific classifications, at least not at such a short distance. The MCLUST results including *z*_strat_ do show lateral variations, but on a more regional scale, which suggests lateral trends of gradually changing properties. This is consistent with information on the geological background, i.e. with different lithostratigraphic units in a wider region that are lateral equivalents. The direction along which these changes occur is, however, not parallel to the layer strike, as one would expect from the known lateral equivalents, but perpendicular to that.

Although the *x*-means *I*_c_ classification only shows two classes (Fig 9), it provides a good indication of the top of the aquitard, and hence is useful for its automatic mapping.

**Fig 11:**
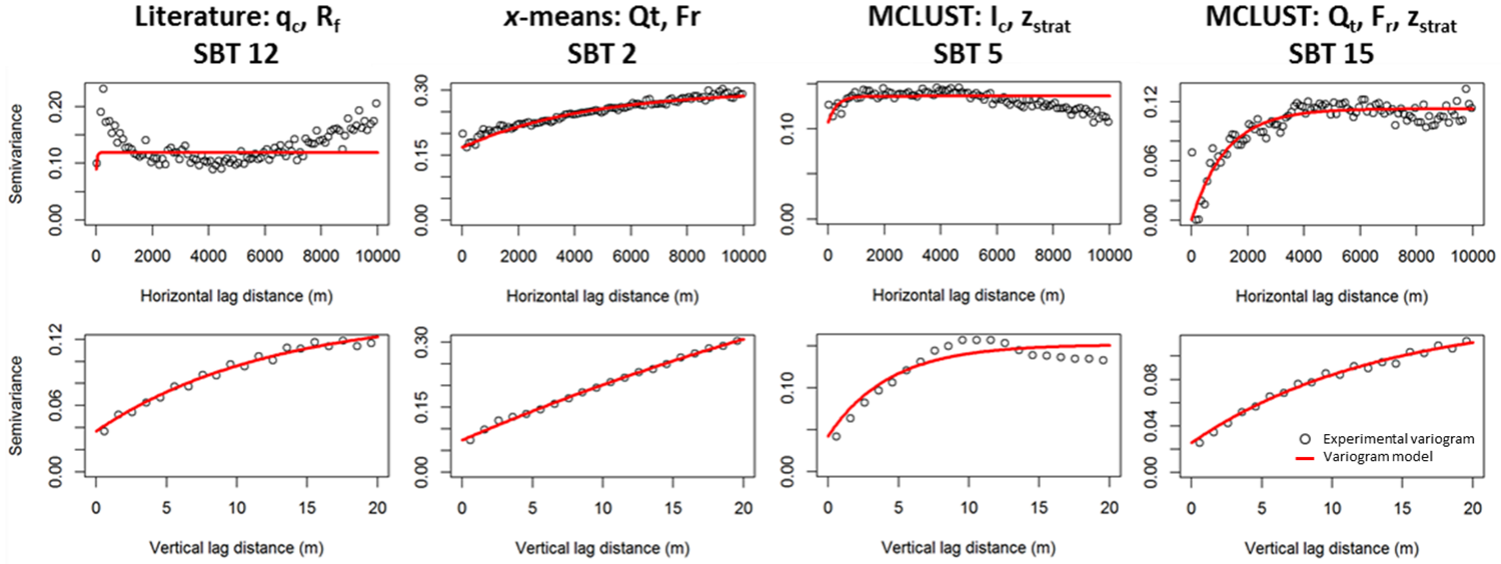
Four examples of typical SBT variograms. The full list of variograms is provided as supplementary material (S1, S2 and S3 Figs).

For a total of 87 SBT indicators encompassing all classifications, variograms were developed. A few typical examples are presented in Fig 11, whereas the full set is provided as supplementary material (S1, S2 and S3 Figs). The first set of variograms for the horizontal and vertical direction is from the literature *q*_c_−*R*_f_ classification (SBT 12): a pure nugget with a hole effect is visible for the horizontal direction. This is due to the splitting of a single lithology type in different SBTs. For example, the random horizontal alternations between SBT 8, 9 and 12 were clearly visible in Fig 10. In the vertical direction spatial correlation is clearly present, meaning that there is at least one section in a large part of the CPTs within the lithostratigraphic column that is consistently classified as SBT 12. As the distance between two CPT points increases, there is an increased chance of transitioning to another SBT.

The second example, based on SBT 2 from the *x*-means classification with *Q*_t_ and *F*_r_ (only 2 classes were obtained, and SBT 2 was shown to be a good indicator of the top of the aquitard), shows an almost linear increase of the semivariance both in horizontal and vertical direction, though the horizontal variogram starts at a very high nugget. This indicates that SBT 2 is predominantly located at a specific depth within the lithostratigraphic column, with the linear shape suggesting that the maximum thickness of that zone is beyond the largest lag distances considered and therefore no plateau is reached in the variogram. Figs 9 and 10 clearly illustrate that this is indeed the case for SBT 2, which predominates at all depths below the top of the aquitard. The horizontal variogram illustrates there is a gradual change in proportion of the SBTs at regional scale.

The third example based on SBT 5 from the MCLUST classification of *I*_c_−*z*_strat_, shows a vertical effective range of 10 to 15 m, and almost a pure nugget in the horizontal direction. This indicates a clearly defined section within the lithostratigraphy, no more than ~10 m thick, which is classified as SBT 5 and which alternates considerably with SBT 6. These classes represent the clayey and sandy parts of the heterogeneous Kasterlee Clay.

The final example is based on SBT 15 from the MCLUST classification with *Q*_t_−*F*_r_−*z*_strat_, and shows a distinct plateau in the semivariance for the horizontal direction. This is an example of the regional-scale lateral changes that has been captured by this SBT classification, and the horizontal range of ~4000 m is a measure for the horizontal extent of the occurrence of SBT 15.

The literature SBT variograms in the supplementary material (S1 Fig) show a mixture of these different variogram types. Pure nuggets or very short ranges occur often in horizontal direction, and the relative nugget values in vertical direction are always high. This indicates that there is considerable random alternation between SBT classes, which is explained by the non-site-specific nature of these classifications. Most *x*-means SBT variograms (S2 Fig) show a linear increase in the vertical direction, and pure nuggets or only a slight increase in horizontal direction, as in the second example discussed above. This indicates the pronounced horizontal and vertical continuity of the *x*-means SBTs, in comparison with all other approaches, and the lack of identification of different lithology types within a single lithostratigraphic unit. The model-based SBT variograms (S3 Fig) consist of a mixture of different types, similar to the literature SBT variograms, but SBTs with a clear horizontal range occur more frequently, indicating the detection of regional lateral changes of the sediment properties. Also, the relative nugget values are generally lower than for the literature SBT variograms, indicating a higher degree of continuity, hence a more robust classification.

**Fig 12:**
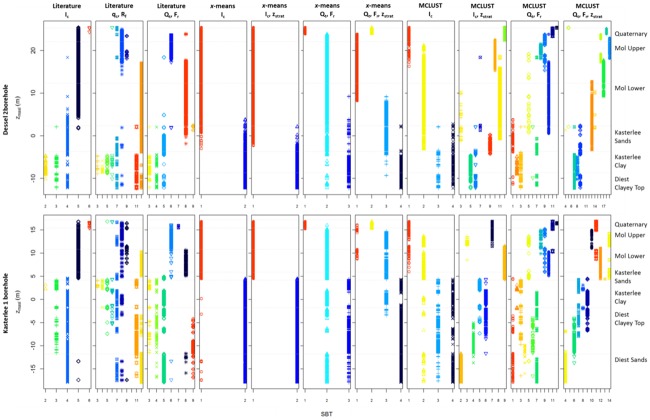
Example SBT logs for the CPT data displayed in Fig 3.

The classification results for the two CPT logs displayed in Fig 3 are presented in Fig 12. The literature *I*_c_ classification does not provide the means to discriminate between the different upper aquifer sands. The difference with the clayey units (Kasterlee Clay and Diest Clayey Top) is however very clear, as is the contrast with the lower aquifer Diest Sands. The literature q_c_−*R*_f_ and *Q*_t_−*F*_r_ classifications show more SBT classes, and allow for a better identification of the different layers. A clear contrast between the upper and lower aquifer is however not always present, and different SBTs are superfluous as their occurrence corresponds exactly to other SBTs (3–5 in the *q*_c_−*R*_f_, and 3–4 in the *Q*_t_−*F*_r_ classification). The *x*-means classifications provide very little information, and only succeed in detecting the top of the aquitard, and the bottom of the Quaternary in some cases. The lower aquifer seems not to be present, as was already clear from Figs 9 and 10. The MCLUST *I*_c_ results provide similar information, but the different units are more clearly identified by including *z*_strat_. For the MCLUST *Q*_t_−*F*_r_ and *Q*_t_−*F*_r_−*z*_strat_ classification, most lithostratigraphic boundaries can be linked to the appearance or dissappearance of certain SBTs or SBT associations (*e*.*g*. SBT 12 and 13 for Mol Lower in the *Q*_t_−*F*_r_−*z*_strat_ classification; SBT 7, 8 and 9 for the Kasterlee Clay and Diest Clayey Top).

### Automated lithostratigraphic mapping

As we are mainly interested in a three-dimensional mapping of the Tertiary lithostratigraphy (from Mol Sands down to Diest Sands), the heterogeneous Quaternary data representing the top stratigraphic layer in the entire area, was discarded prior to the mapping analysis. This avoids interference of these data in the automatic detection of layer boundaries. The upper 3 m of each individual CPT test was removed, as the average depth of the Quaternary in the cored boreholes is ~3 m.

**Fig 13:**
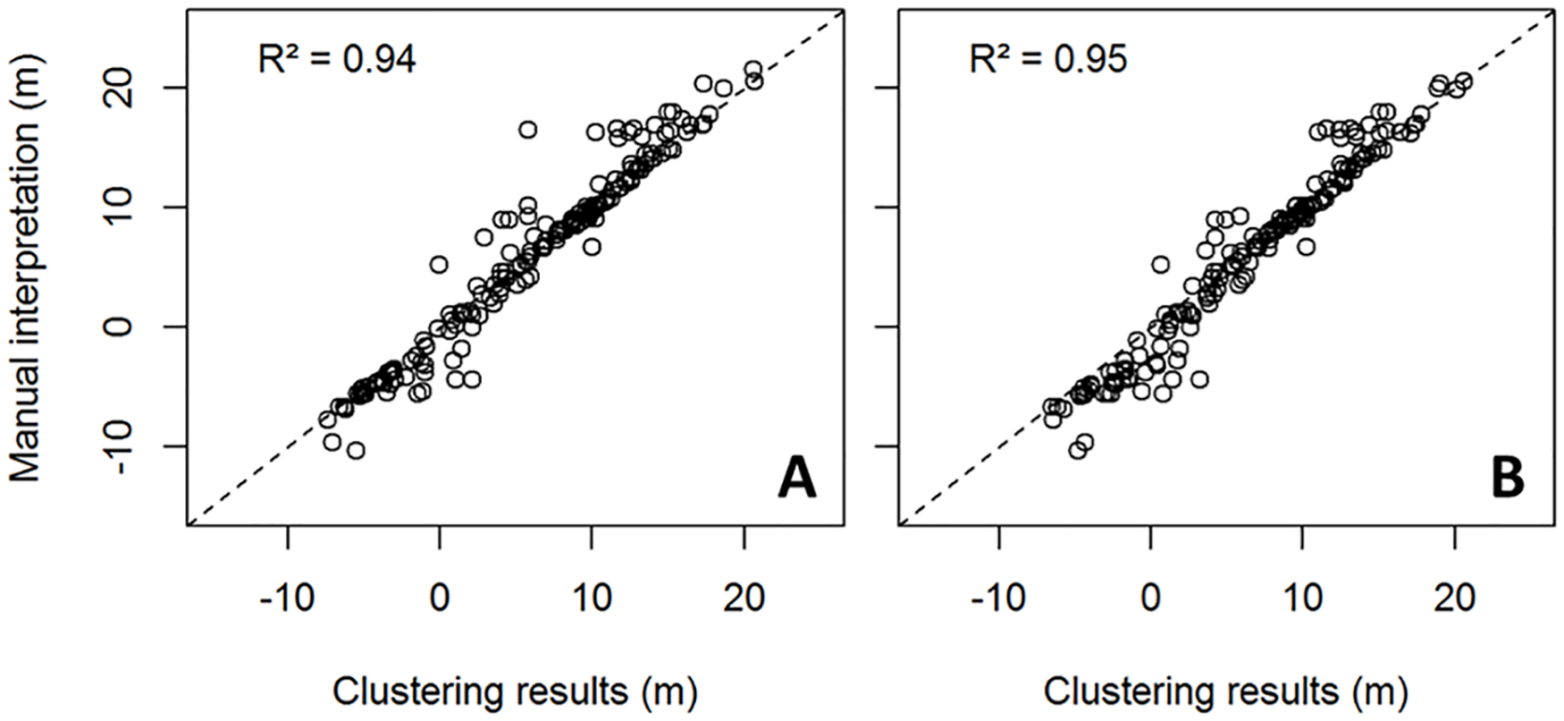
Scatterplot of lithostratigraphic mapping of the top of the aquitard (in m below sea level) versus the manually interpreted top of the aquitard [17, 18], using A) *x*-means clustering and B) model-based clustering.

The *x*-means and MCLUST algorithms are applied to the *I*_c_ data to obtain two SBT classes. For the *x*-means classification, this corresponds exactly to the results previously discussed, as the use of 2 SBTs was most optimal according to the BIC. The results of using the kernel density estimates of *z*_masl_ to pinpoint the top of the aquitard are plotted in Fig 13 versus the manually interpreted depth values by Schiltz [17, 18], as explained above. Both approaches show a reasonably good correspondence, with R² values of 0.94 and 0.95 for respectively the *x*-means and model-based clustering. The maximum deviation amounts to 10.8 and 7.4 m, with ~60% of the data within 0.6 and 1 m of the manually identified boundary and 25% within 0.18 and 0.26 m. The largest differences occur at the outer boundary of our study area. We believe that the reason for these differences is the manual interpretation which can account for nearby CPT data, while the automated mapping approach considers a single CPT test at a time.

**Fig 14:**
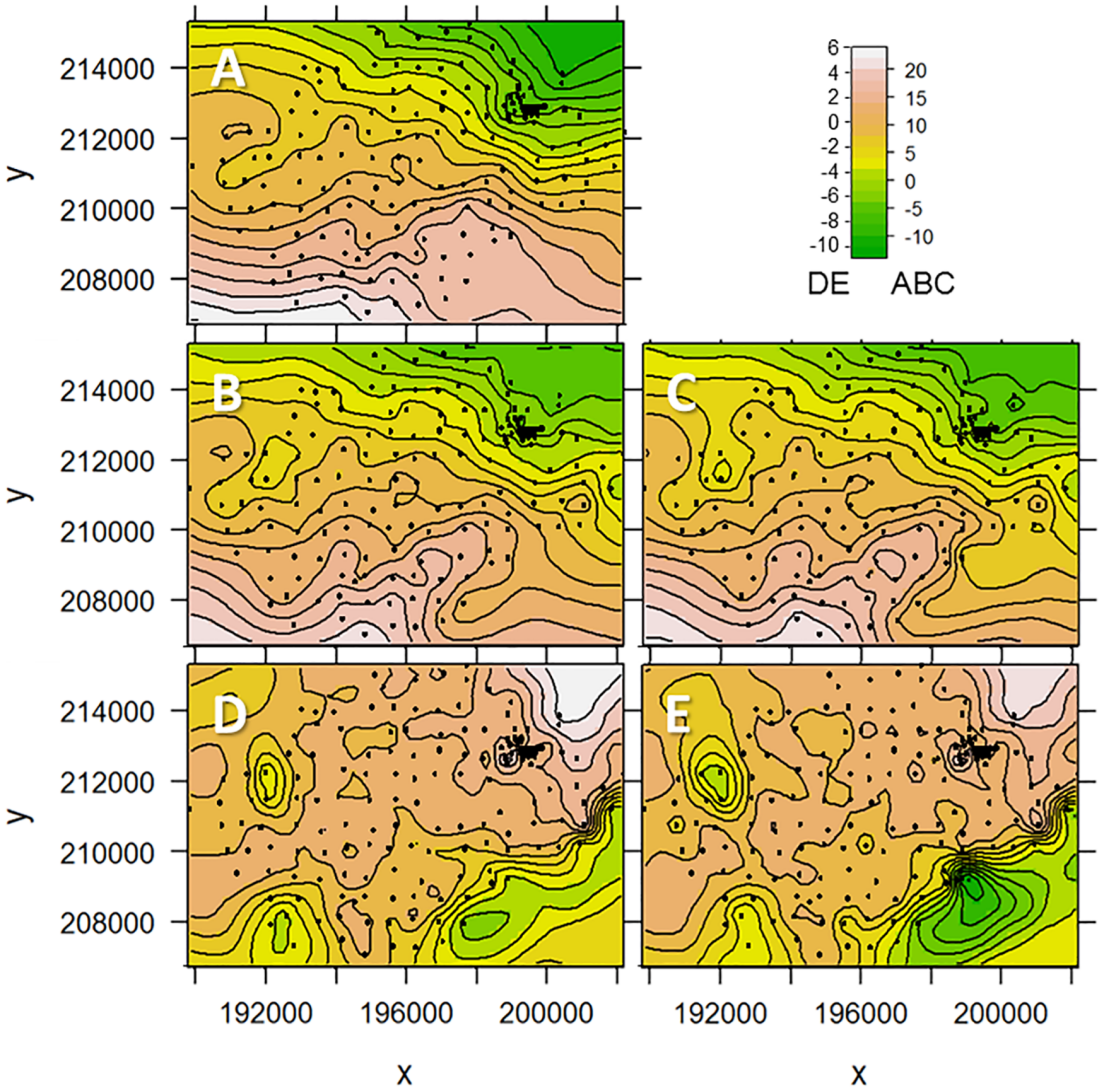
Contour maps of the top of the aquitard, using A) a manual approach, B) the model-based and C) the *x*-means clustering. Differences between the automatically and the manual derived reference values are presented in D) for the model-based and E) for the *x*-means clustering. Locations outside of the CPT characterization area are influenced by extrapolation.

The contour maps resulting from universal kriging of the identified boundary locations are shown in Fig 14. The main misfit between the manually and automatically interpreted boundaries occurs at the southern border of the study area. In this area, the upper aquifer is only a few meters thick, and hence mapping of the top of the aquitard is more difficult than in the other regions. As the manual interpretation also accounted for i) identified depth locations in nearby CPTs, and ii) the general trend of the aquitard top dipping in NE direction, the manual interpretation is probably more accurate and therefore it was used in the previously discussed clustering approaches to obtain *z*_strat_. On the other hand, the automatic approach is more objective than the manual approach, and consistently always uses the same criterion for detecting the lithostratigraphic boundary. More detailed investigations, *e*.*g*. cored boreholes, are needed to discriminate between both approaches in the areas with the largest misfit.

## Conclusion

We have shown that model-based SBT classifications of CPT data can be useful for regional lithostratigraphic mapping. The obtained SBT classes provide more detailed information than those obtained with frequently used deterministic unsupervised clustering algorithms like *k*- and *x*-means clustering. Moreover, the obtained classification better honours the intrinsic classes within the data, in contrast to the classical literature SBT classification charts. These findings were further corroborated by considering the multivariate sediment properties from cored boreholes in combination with the SBT classes, and by studying the spatial distribution of the obtained classes. The derived SBT classes where shown to be correlated with class-average sediment properties such as clay content, density, porosity, etc. Such relationships may be used to provide estimates of physical and hydraulic properties at a regional scale. The use of the stratigraphic depth for clustering proved to be useful for the presented case study, and is recommended for geologically layered sites (unconsolidated sedimentary rocks).

We also proposed a new methodology for automated lithostratigraphic mapping using site-specific SBTs, which was applied to map the top of an aquitard in a regional CPT dataset. Comparison with the more traditional time-consuming manually interpreted results for the top of the aquitard suggests that this methodology can be very useful in practice, on its own, or to support manual interpretation based on the literature SBT classifications that provide indications on lithology, but lack information on the true typology of the data. When dealing with a layered stratigraphy, or distinct sedimentary bodies, this approach is useful to delineate different geological/geotechnical features. The automated mapping was only tested on a single boundary within the lithostratigraphic column (*i*.*e*. for the top of an aquitard). Further research should address the joint mapping of different boundaries and layers. Moreover, to make the identification of the boundaries more robust, a probabilistic approach for locating a horizon might be useful. Together with the spatial correlation of the horizon elevation, this could result in more robust regional estimates of the horizon elevation (*e*.*g*. through kriging accounting for measurement error). Another approach for increasing the robustness might be the use of airborne geophysics, as recently demonstrated by Friedel [62] with borehole data. Furthermore, the inclusion of a priori knowledge on the layer geometries could be included as well, in a Bayesian setting.

## Supporting information

S1 Fig Overview of the literature classification variograms. (TIF)

S2 Fig Overview of the x-means classification variograms. (TIF)

S3 Fig Overview of the MCLUST classification variograms. (TIF)
