# Supplementary figures and images for: Model-based classification of CPT data and automated lithostratigraphic mapping for high-resolution characterization of a heterogeneous sedimentary aquifer
Source: PLoS One. 2017 May 3;12(5):e0176656. doi: 10.1371/journal.pone.0176656 (PMC5415173; doi:10.1371/journal.pone.0176656)

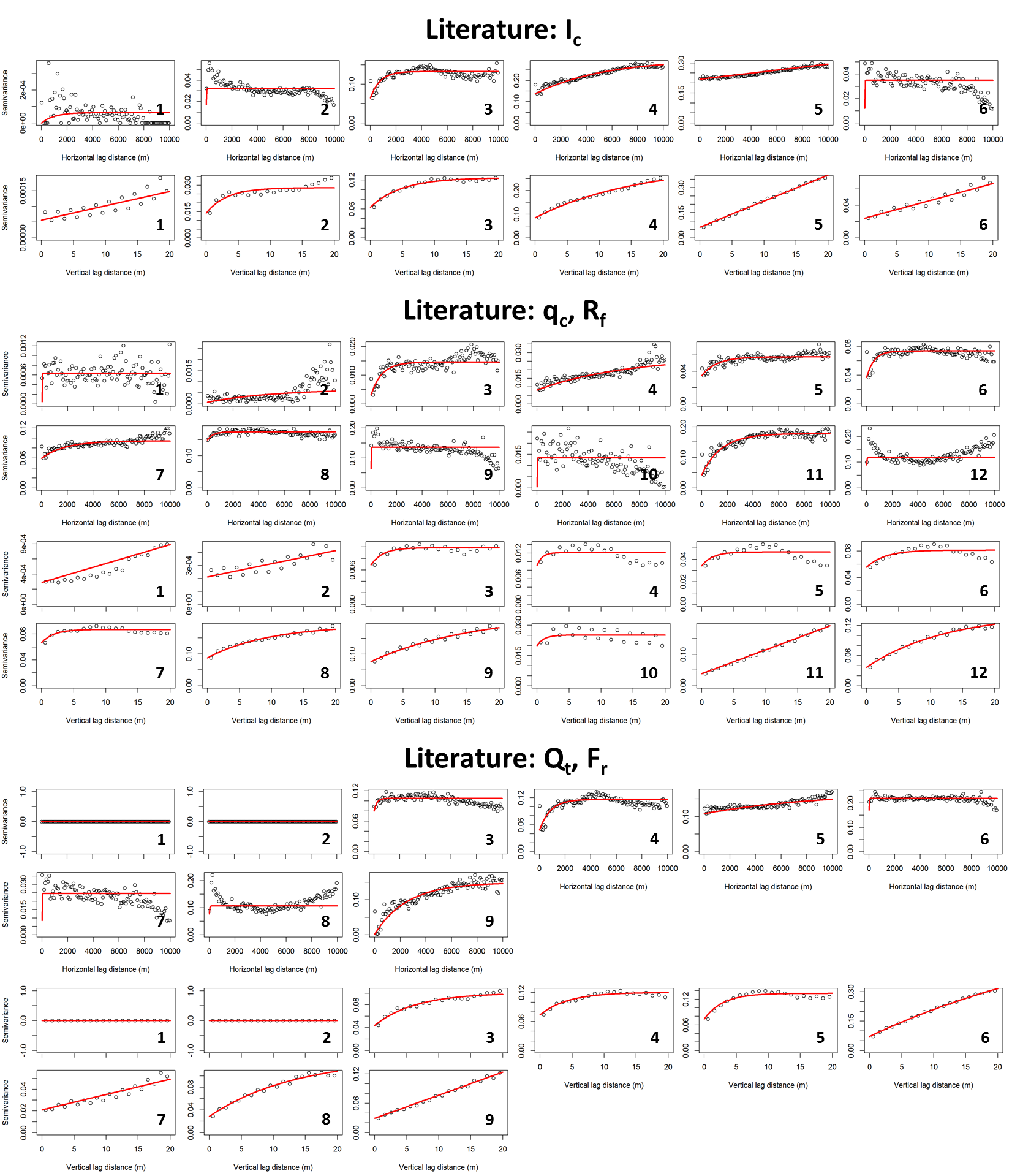

Supplement: S1 Fig — (TIF) [file pone.0176656.s001.tif]

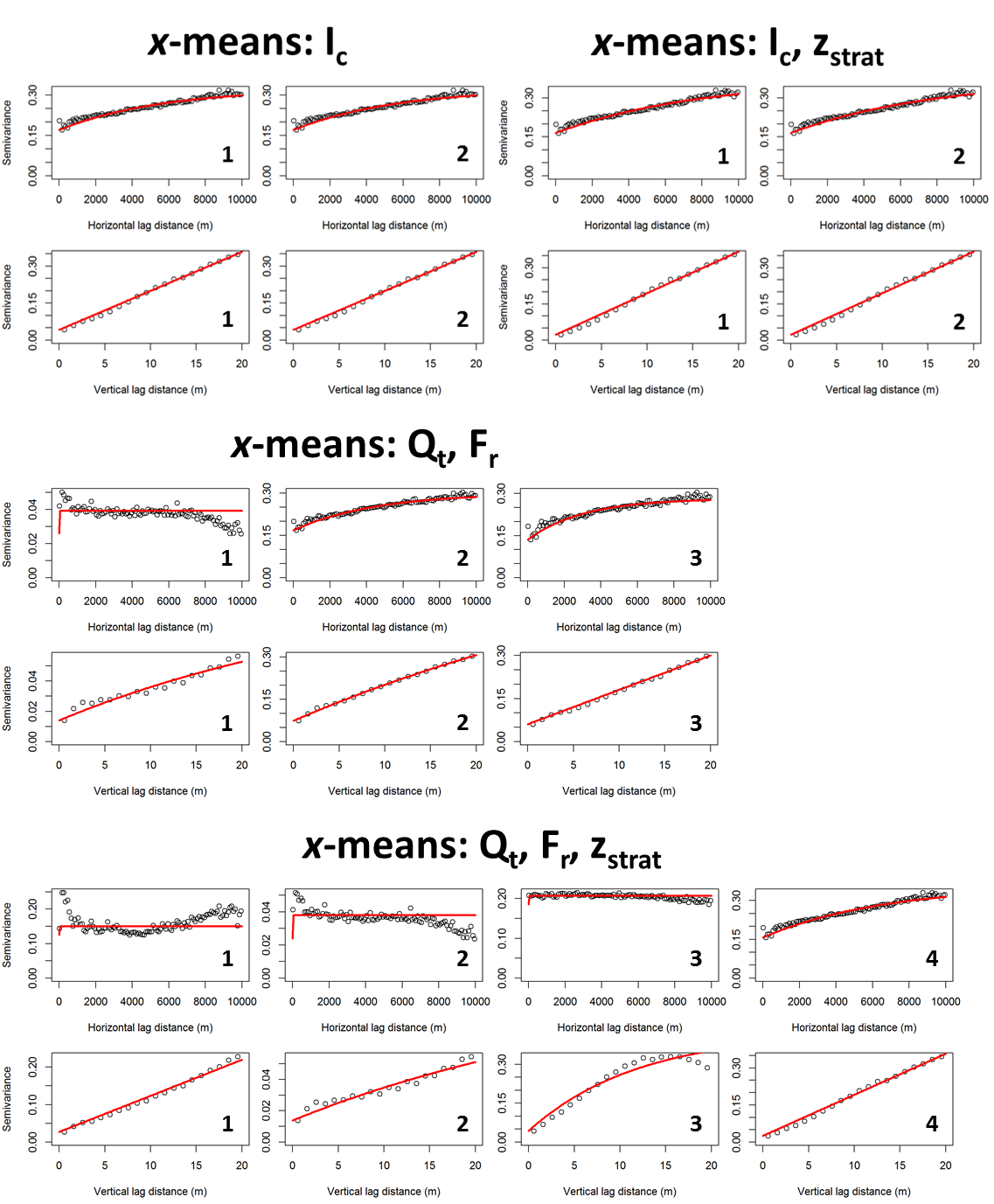

Supplement: S2 Fig — (TIF) [file pone.0176656.s002.tif]

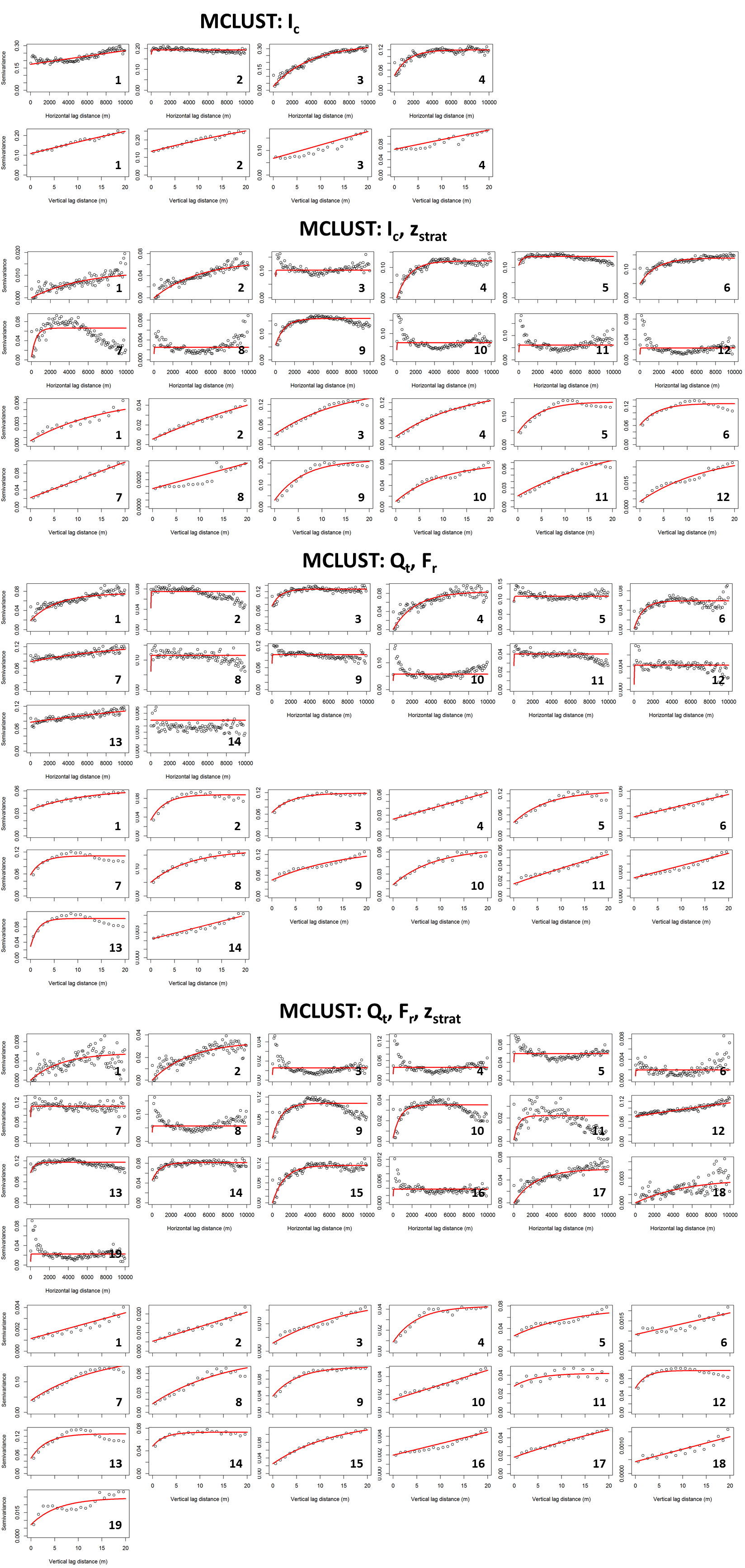

Supplement: S3 Fig — (TIF) [file pone.0176656.s003.tif]
